# Characteristics of Patients Involved With the Kidney Supportive Care Program: A Cohort Study of Routinely Collected Data

**DOI:** 10.1016/j.xkme.2026.101470

**Published:** 2026-07-15

**Authors:** Andrew Jeyaruban, Sarah A. Roxburgh, Sara J. Rendo, Daniel V. O’Hara

**Affiliations:** 1 Department of Renal Medicine, Royal North Shore Hospital, New South Wales, Australia; 2 Renal and Metabolic Division, The George Institute for Global Health, The University of New South Wales, New South Wales, Australia; 3 Northern Clinical School, Faculty of Medicine and Health, The University of Sydney, New South Wales, Australia; 4 Sydney Clinical Trials Centre, Faculty of Medicine and Health, The University of Sydney, New South Wales, Australia

**Keywords:** Kidney supportive care, conservative kidney management, survival, palliative care, nondialytic management

## Abstract

**Rationale & Objective:**

People with kidney failure face a significantly higher risk of death and symptom burden. We sought insights into the survival and symptom burden of people receiving medical management of kidney failure without dialysis.

**Study Design:**

Retrospective cohort study.

**Setting & Participants:**

People with an estimated glomerular filtration rate (eGFR) ≤ 15 mL/min/1.73 m^2^ receiving nondialytic/conservative/palliative management and known to the Kidney Supportive Care Team in a tertiary care hospital.

**Predictors:**

eGFR at cohort entry, living arrangement, and history of ischemic heart disease, heart failure, or stroke, according to routine care databases.

**Outcomes:**

Primary: median survival. Secondary: number of hospitalizations and symptom burden assessed through the Integrated Palliative Care Outcome Scale-Renal score.

**Analytical Approach:**

Cox regression survival modeling and descriptive statistics for hospitalizations and symptom burden.

**Results:**

The cohort included 223 people receiving care between January 2016 and April 2023, with a median age of 84 (interquartile range, 77-87) years. Median survival was 452 days (95% CI, 351-552). People with an eGFR of 11-15 mL/min/1.73 m^2^ at cohort entry had a median survival of 517 days (95% CI, 406-623); those with an eGFR of 6-10 mL/min/1.73 m^2^ had a median survival of 260 days (95% CI, 138-382), whereas people with an eGFR of < 5 mL/min/1.73 m^2^ had a median survival of 61 days (95% CI, 46-107). People living independently at home had significantly higher median survival (538 days), compared with assisted living at home (356 days) or living in a nursing home (232 days) (*P* < 0.05). The studied comorbidities were not predictive of survival. At year 1, the most prevalent symptom was weakness (60%) with median “moderate” severity, with similar findings at year 2.

**Limitations:**

Single-center, retrospective study with univariable analysis.

**Conclusions:**

The study provided valuable insights into life expectancy and symptom burden with kidney failure managed without dialysis, including the prognostic value of considering the baseline living situation in anticipating survival, to help guide clinicians facing the uncertainty of this clinical scenario.

## Plain Language Summary

This study explored the outcomes of people with kidney failure managed without dialysis in a single Australian center through medical records. The study was undertaken to improve knowledge about survival expectations and symptom burden in people with kidney failure. In this study, survival varied between individuals, but half of the participants survived for 15 months. People with better kidney function tended to live longer, as did people living at home independently compared with people requiring living assistance or residing in a care facility. The most common symptom seen was weakness, affecting 6 out of every 10 people. The data from the study will enable us to have more informed conversations with people who are approaching kidney failure and their families.

## Introduction

People with kidney failure face a higher risk of death and symptom burden than their peers,^1^^,^^2^ and may experience a poorer quality of life.^3^, ^4^, ^5^, ^6^ The impact of symptoms and the impairment of quality of life have been likened to people experiencing a malignancy.^7^^,^^8^ Most studies exploring quality of life and symptom control in people with kidney failure have examined those receiving hemodialysis rather than those opting for a conservative treatment pathway.^3^^,^^5^ Conservative management is associated with a significantly higher mortality risk, with people commencing dialysis showing 2.3 times longer median survival,^9^ although survival rates vary substantially between studies.^9^, ^10^, ^11^, ^12^ Survival benefits are less clear in older people, particularly those ≥85 years,^9^ or in those ≥80 years with comorbidities.^9^^,^^10^ As such, for many people, medical management without dialysis is an appropriate treatment decision, particularly when supported by a kidney supportive care (KSC) service.^13^ KSC is a comprehensive conservative kidney management model that includes interventions to delay progression of kidney disease, management of symptoms, and detailed communication, including advance care planning, while providing psychological, social, and family support.^14^ The focus is to prioritize quality of life in keeping with people’s goals of care.^8^^,^^15^ A study undertaken in Australia revealed that up to 20% of people with kidney failure referred to a nephrologist had followed a conservative kidney management pathway.^14^

It can be difficult to counsel people on what to expect for their future health trajectory because of the unpredictability of kidney failure, the variation in survival rates in previous studies, and the lack of clarity about which baseline factors are predictive of a survival difference. To aid these discussions, we aimed to explore the survival and symptom burden of people receiving medical management of kidney failure who were under the care of a KSC team.

## Method

This was a retrospective cohort study of people known to the KSC team in a tertiary metropolitan nephrology unit in Australia. The study used data routinely collected as part of clinical care. The study included all people in the KSC database from January 1, 2016, to April 30, 2023, involving those reviewed in inpatient and clinic settings with an eGFR of ≤ 15 mL/min/1.73 m^2^ on conservative kidney management or those who were undecided for at least 6 months before the commencement of the study. The KSC database includes participants’ basic demographic characteristics, the latest eGFR at each contact with the service, and the results of routine symptom scoring with the Integrated Palliative Care Outcome Scale (IPOS)-renal questionnaire.^16^ It has been maintained by the KSC clinical nurse consultant as well as the team registrars over the last 7 years. Further data, including comorbidities, living situation, hospitalizations, deaths, place of death, and records of Advanced Care Directives or formal discussions of future care preferences, were obtained from the electronic medical record through manual chart review.

The patients’ eGFR eligibility was confirmed at least 1 month later to ensure those with acute kidney injury were not included in the study. People were excluded if they had active malignancy (other than nonmelanoma skin cancer), if they were receiving kidney replacement therapy, or if there were inadequate data for kidney function (at least 2 readings from 2 separate dates were required).

The primary outcome of the study was median survival in days. The secondary outcomes were hospitalizations, and symptom burden scored using the routinely administered IPOS-renal questionnaire, with a range of 0-60 for physical symptoms, with a higher score indicating worsening symptom burden.^16^ Secondary outcomes also included preferred versus actual place of death, determined by reviewing the advance care directive when completed, and comparing it to the recorded place of death.

The SPSS program (version 22) was used for statistical analysis.^17^ Median survival was assessed according to predetermined baseline predictors through Cox regression modeling. The survival analyses were stratified according to comorbidities that were commonly associated in patients with kidney failure in clinical practice, including by eGFR category at entry into our database, by living situation (independent living vs assisted living at home vs living in nursing home), by selected comorbidities (the presence or absence of ischemic heart disease, heart failure, stroke, diabetes, or chronic liver disease), by the presence of proteinuria at baseline, and by the use of angiotensin-converting enzyme inhibitors or angiotensin receptor blockers. Descriptive statistics were used to explore hospitalizations and symptom burden.

The project was approved by the Northern Sydney Local Health District Human Research Ethics Committee (approval no: 2023ETH02054). Informed consent was waived because of the use of deidentified information.

## Results

### Baseline Characteristics

There were 736 patients in the KSC database. This included all people referred to the service from January 1, 2016, to April 30, 2023. Following exclusions, 223 people were included in the final analysis (Fig 1 depicts the cohort inclusion flow). Reasons for exclusion included use of kidney replacement therapy (331 people), history of active malignancy (35), acute kidney injury or a baseline eGFR of > 15mL/min/1.73 m^2^ (140), change of modality from conservative to kidney replacement therapy (4), or a lack of pathology results (3).

**Figure 1:**
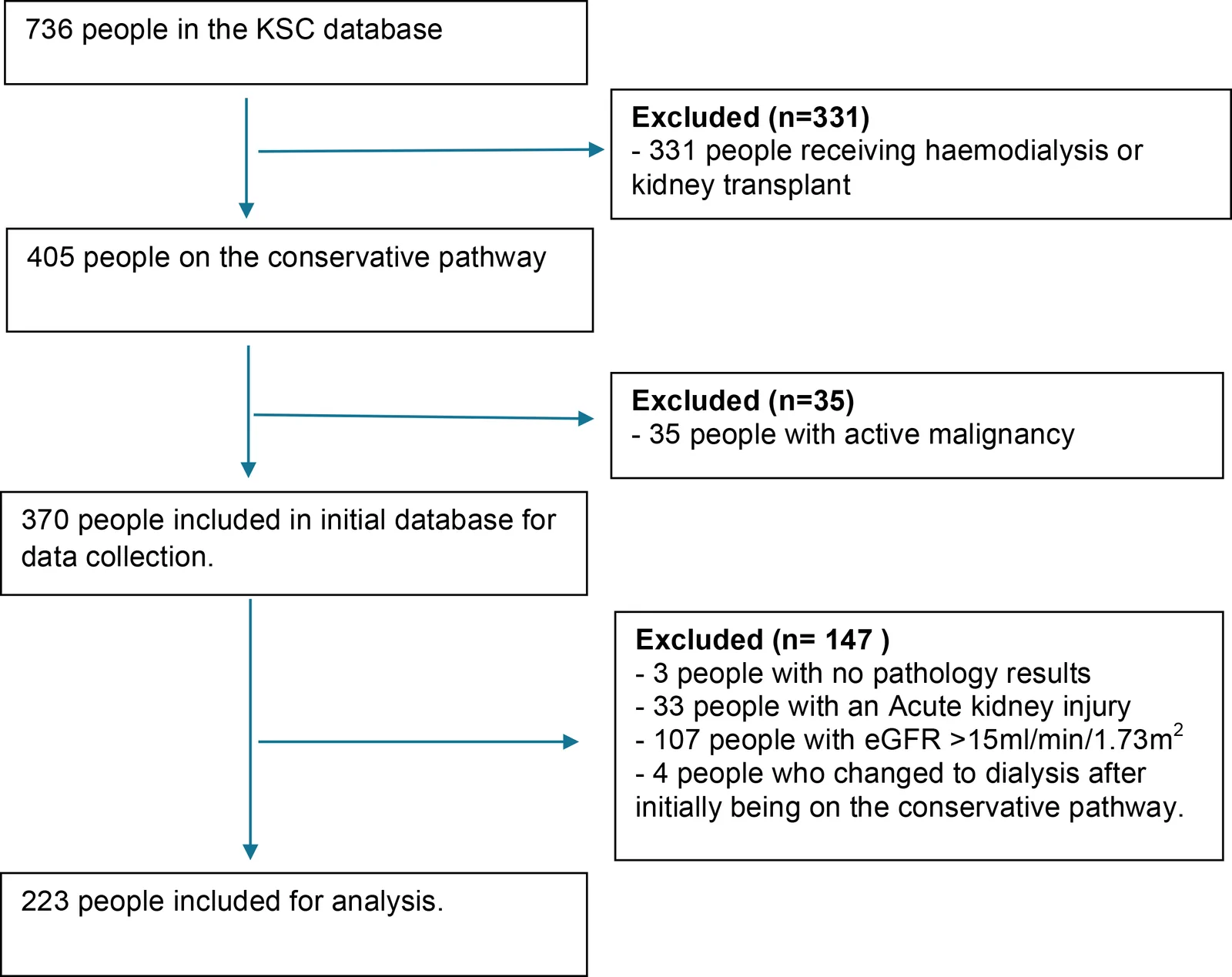
Flow chart of people included in the study. Abbreviations: KSC, kidney supportive care; eGFR, estimated glomerular filtration rate

The median age was 84 (interquartile range [IQR], 77-87) years (Table 1), and 85 people (38%) were female. The majority were in the eGFR range of 11-15 mL/min/1.73 m^2^ (n=182, 81.6%) on entry into the cohort, followed by 39 people (17.5%) in the range of eGFR 6-10 mL/min/1.73 m^2^ and 2 people (0.9%) with an eGFR ≤ 5 mL/min/1.73 m^2^.

**Table 1:** Baseline Characteristics of People in the Study

Variable	Value
Age, y, median (interquartile range [IQR])	84 (77-87)
eGFR at entry, n(%)	
11-15 mL/min/1.73 m^2^	182 (81.6)
6-10 mL/min/1.73 m^2^	39 (17.5)
< 5 mL/min/1.73 m^2^	2 (0.9)
Serum albumin baseline level, mg/mmol, median (IQR)	34 (30-38)
Proteinuria baseline, mg/mmol, median (IQR)	259 (95-509)
Sex^a^	
Male, n (%)	138 (61.9)
Female, n (%)	85 (38.0)
Ischemic heart disease, n (%)	51 (22.9)
Heart failure, n (%)	103 (46.2)
Peripheral vascular disease, n (%)	35 (15.7)
Diabetes, n (%)	106 (47.5)
Stroke, n (%)	33 ( 14.8)
Dementia, n (%)	1 (0.4)
Chronic liver disease, n (%)	10 (4.5)
Advance care directive completed, n (%)	123 (51.5)
ACEI/ARB use	65 (29.1)
Living situation	
Independent, n (%)	138 (61.3)
Requires assistance, n (%)	56 (24.9)
Nursing home, n (%)	23 (10.2)

Abbreviations: ACEI, angiotensin-converting enzyme inhibitor; ARB, angiotensin receptor blocker; eGFR, estimated glomerular filtration rate; IQR, interquartile range.

a As documented in the patient's medical record.

Nearly half of all patients had a diagnosis of heart failure (103 people, 46.2%) or diabetes (106 people, 47.5%), whereas 35 people had peripheral vascular disease (15.7%) and 33 had experienced a previous stroke (14.8%). The majority of people on entry to the database were independent with activities of daily living (n=138, 61.3%), whereas 56 people (24.9%) lived at home and needed assistance, and 23 (10.2%) people lived in a nursing home (Table 1).

### Survival

Among the 223 participants, 176 died during the follow-up period (78.9%, Table 2). The overall median survival of the cohort was 452 days (95% confidence interval [CI], 351-552). People with an eGFR of 11-15 mL/min/1.73 m^2^ on entry to the database had a median survival of 517 days (95% CI, 406-628); people with an eGFR of 6-10 mL/min/1.73 m^2^ had a median survival of 260 days (95% CI, 138-382); and people with an eGFR of ≤ 5 mL/min/1.73 m^2^ had a median survival of 61 days (95% CI, 46-107) (*P* < 0.05) (Table 2 and Fig 2 ).

**Table 2:** Patient Outcomes: Survival (Days), Death, and Hospitalizations

Category	Median Survival (d)	n (%)	*P* value
Survival	452 (95% CI 351-552)	—	—
eGFR 11-15mL/min/1.73 m^2^	517 (95% CI, 406-628)	182 (81.6)	< 0.05
eGFR 6-10 mL/min/1.73 m^2^	260 (95% CI, 138-382)	39 (17.5)	
eGFR ≤ 5 mL/min/1.73 m^2^	61 (95% CI, 46-107)	2 (0.9)	
Independent living	538 (95% CI, 330-746)	138 (61.9)	< 0.05
Assisted living at home	356 (95% CI, 262-450)	56 (25.1)	
Living in nursing home	232 (95% CI, 80-384)	23 (10.3)	
Ischemic heart disease	434 (95% CI, 277-591)	51 (22.9)	0.30
No ischemic heart disease	475 (95% CI, 378-572)	172 (77.1)	
Heart failure	367 (95% CI, 289-445)	103 (46.2)	0.17
No heart failure	538 (95% CI, 359-717)	120 (53.8)	
Stroke	472 (95% CI, 239-704)	33 (14.8)	0.47
No stroke	452 (95% CI, 346-558)	190 (85.2)	
Deaths	—	176 (78.2)	—
eGFR 11-15 mL/min/1.73m^2^	—	141 (77.5)	—
eGFR 6-10 mL/min/1.73m^2^	—	33 (84.6)	—
eGFR ≤ 5 mL/min/1.73m^2^	—	2 (100.0)	—
Independent living	—	104 (75.4)	—
Assisted living at home		50 (89.3)	
Living in nursing home		19 (82.6)	
Hospitalizations		399	
Hospitalizations (by eGFR at cohort entry)			
eGFR 11-15 mL/min/1.73 m^2^		333	
eGFR 6-10 mL/min/1.73 m^2^		62	
eGFR ≤ 5 mL/min/1.73 m^2^		4	
Hospitalizations (by episodes per patient)			
0		40 (17.9)	
1		63 (28.3)	
2		69 (30.9)	
3		27 (12.1)	
4		13 (5.8)	
5		19 (4)	
6		1 (0.4)	
14		1 (0.4)	

Abbreviations: CI, confidence interval; eGFR, estimated glomerular filtration rate.

**Figure 2:**
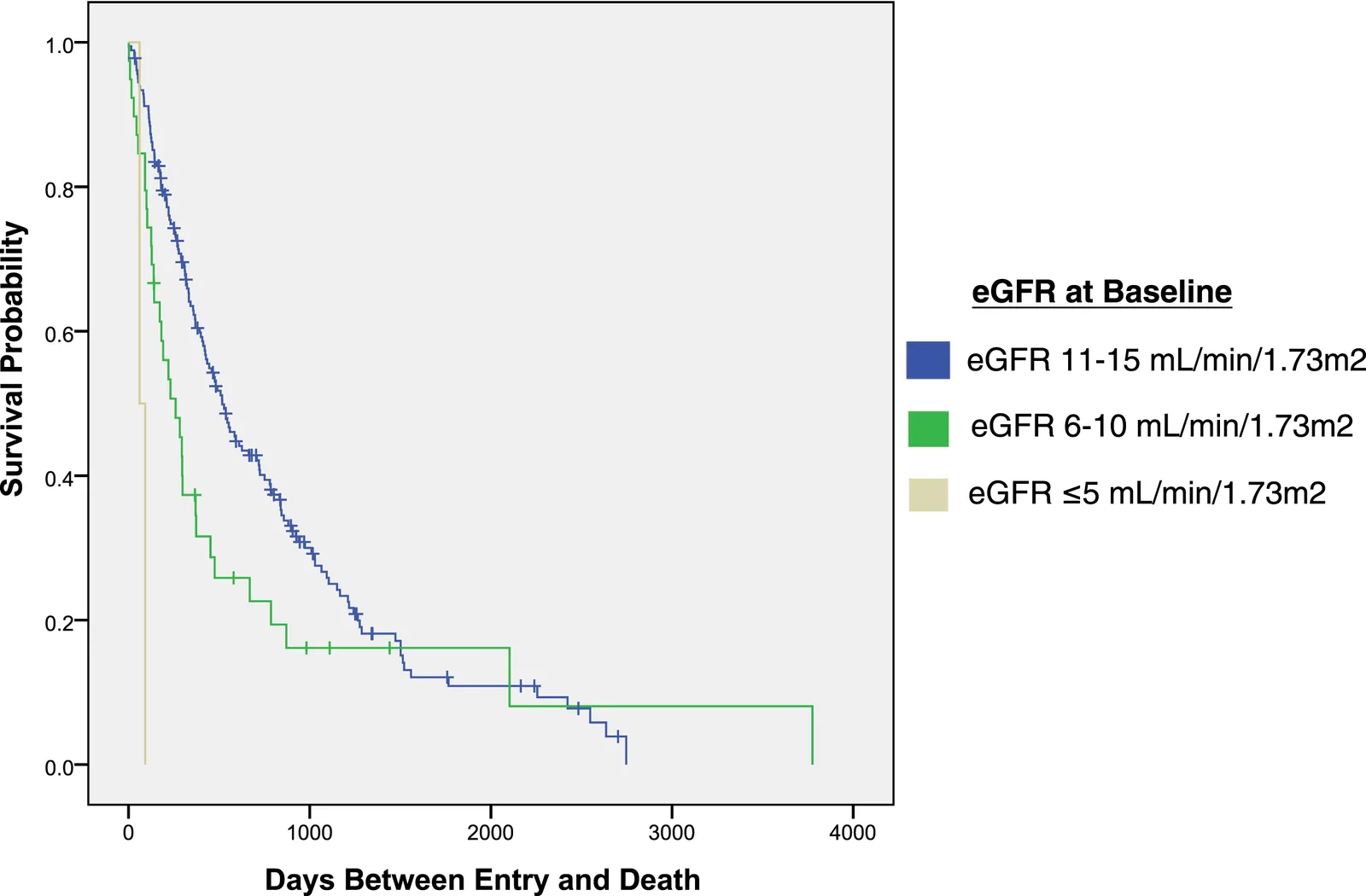
Kaplan-Meier curve revealing the association between eGFR category at baseline and survival. Abbreviations: eGFR, estimated glomerular filtration rate.

People living independently at home had statistically greater survival (median 538 days), compared with those assisted living at home (median 356 days) or living in a nursing home (median 232 days; *P* ≤ 0.05) (Table 2).

There was no statistically significant association with longer survival in people with a history of ischemic heart disease versus no ischemic heart disease (434 vs 475 days, *P* = 0.3) or by the presence or absence of heart failure (367 vs 538 days, *P* = 0.17), peripheral vascular disease (545 vs 445 days, *P* = 0.297), stroke (472 vs 452 days, *P* = 0.47), diabetes (490 vs 423 days, *P* = 0.53), or chronic liver disease (490 vs 445 days, *P* = 0.74) in unadjusted analyses. A cardiovascular composite of previous heart failure, ischemic heart disease, and stroke was not significantly associated with survival (472 vs 452 days, *P* = 0.39). The prescription of ACE inhibitors or angiotensin receptor blockers (538 vs 370 days, *P* = 0.12) or the presence versus absence of proteinuria (490 vs 323 days, *P* = 0.47) was not statistically significantly associated with survival.

### Symptom Score

There were 135 people with baseline IPOS-renal scores compared with 42 people with IPOS-renal scores after 1 year, 11 people after 2 years, and 3 people after 3 years of follow-up. The median IPOS-renal score at baseline was 11 (IQR, 6-16, noting that the IPOS-renal symptom score has a potential range of 0-60), at year 1 was 13 (IQR, 8-17), and at year 2 was 14 (IQR, 8-20) (Fig 3). The median symptom burden was numerically greater among those with an eGFR of 6-10 mL/min/1.73 m^2^ than among those with an eGFR of 11-15 mL/min/1.73 m^2^, although there were no baseline IPOS-renal scores recorded for those with eGFR ≤ 5 mL/min/1.73 m^2^ (Table 3).

**Figure 3:**
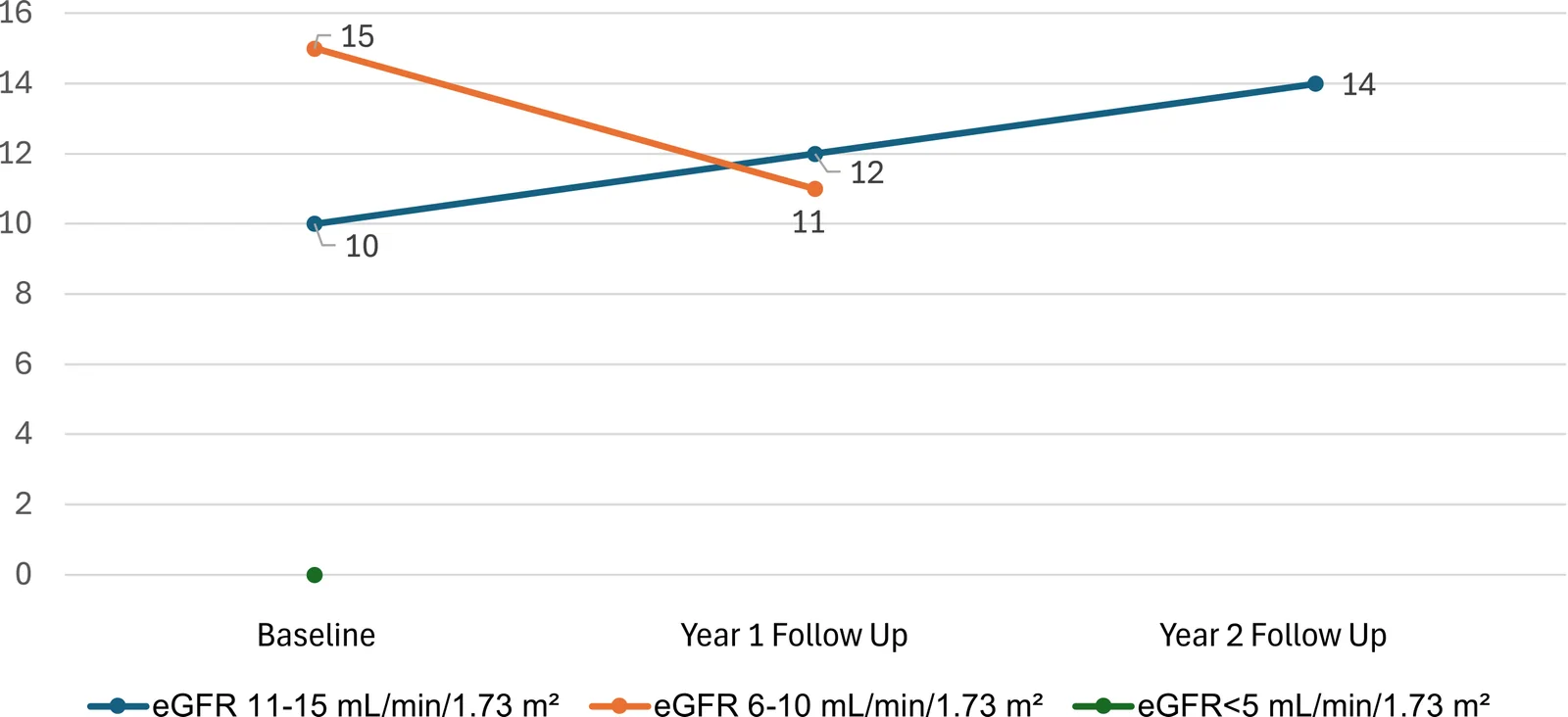
Median IPOS-renal symptom scores at baseline and after 1 and 2 years of follow-up based on baseline eGFR category on entry to the database.Abbreviations: eGFR, estimated glomerular filtration rate; IPOS-renal, Integrated Patient Outcome Scale-Renal.

**Table 3:** The Symptom Scores (Median IPOS-Renal Score) of People in the Study Compared with the Baseline eGFR on Entry There were no people with IPOS-renal scores with entry eGFR less than 5 mL/min/1.73 m^2^

Baseline Kidney Function	Baseline	Year 1	Year 2
eGFR 11-15 mL/min/1.73 m^2^	—	—	—
Median (IQR)	10 (6-16)	12 (9-18)	14 (8-20)
n	114	40	11
eGFR 6-10 mL/min/1.73 m^2^	—	—	—
Median (IQR)	15 (8-18)	11 (9-13)	0
n	21	2	0

Abbreviations: eGFR, estimated glomerular filtration rate; IPOS, Integrated Palliative Care Outcome Scale; IQR, interquartile range.

The most prevalent symptom at baseline was weakness in 80% of people (n=108), with a median “moderate” severity, followed by poor mobility (n=100, 74%) with a median severity of “moderate.” At year one, the most prevalent symptoms reported were weakness at 60% (n=81) and poor mobility at 57% (n=77), with a median “moderate” severity, followed by pruritus at 33% (n=46) with a median “moderate” severity. At year 2, weakness and poor mobility remained the most prevalent at 64% (n=27) and 45%, respectively (n=19), followed by pain at 29% (n=12), all of which had a median “moderate” severity. There was no association between delta eGFR (calculated by dividing the difference between entry and final eGFR over the duration of follow-up for each individual) and median IPOS-renal scores (*P* = 0.75) or between total IPOS-renal scores and survival (*P* = 0.25).

### Hospitalization

There were a total of 339 hospitalizations in this cohort. The median number of hospitalizations per person was 2, with a range of 0-14. Forty people had no hospitalizations, 181 people had 1-5 hospitalizations, and 2 people had > 5 hospitalizations (Table 2).

### Advance Care Directives and Place of Death

Advance care directives (ACD) were completed by 123 people over the duration of the study (55.2%). The ACD template includes the option to express a preferred location for end-of-life care. The most common answer was that individuals were unsure of their preferred location (40, comprising 32.5% of those who had completed an ACD). A further 37 people wanted to have end-of-life care at home (30.1%), compared with 19 people preferring care in the palliative care unit (15.4%), 16 in the hospital (13.0%), and 11 in the nursing home (8.9%).

Among the 176 people who died, 84 deaths occurred in the hospital (47.7% of deaths). Thirty-nine people died in the palliative care unit (22.1%) compared with 29 in the nursing home (16.4%) and 21 at home (11.9%). Among people with an ACD, 39 patients out of 123 (31.7%) died in the hospital, whereas 45 out of 85 (52.9%) people without an ACD died in the hospital (Fig 4).

**Figure 4:**
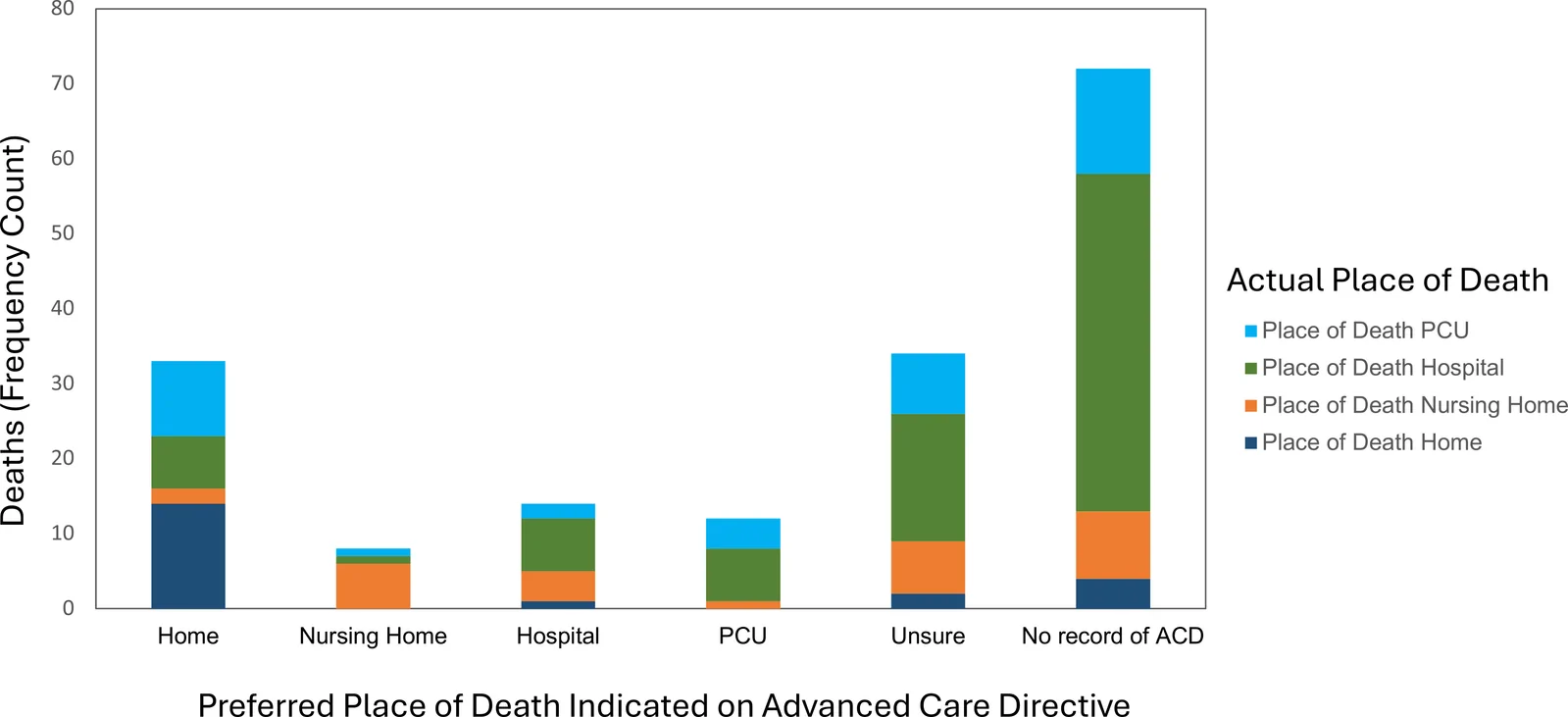
Place of death versus preferred place of death as indicated on the advanced care directive (ACD). Abbreviations: PCU, palliative care unit.

## Discussion

This study explored the survival and symptom burden of people with conservatively managed kidney failure through a retrospective cohort database study of patients known to the KSC team at a metropolitan tertiary hospital. The study revealed that overall survival ranged from over a year among those entering the database with an eGFR of 11-15 mL/min/1.73 m^2^, to 2 months among people with entry to the database with an eGFR of < 5 mL/min/1.73 m^2^. There was a significant association between a higher entry eGFR and better survival. However, there was no significant association between survival and the studied comorbidities. Living independently at the database entry was associated with better survival. The most common symptom at baseline and follow-up was weakness, followed by poor mobility. There was no significant association between IPOS-renal scores and survival.

Median survival varies widely in published cohorts of people with conservatively managed kidney disease, from 1 to 41 months in a meta-analysis of 21 cohort studies.^11^ Variation in baseline kidney function, cohort age distribution, comorbidity burden, and care provision likely contribute. In another retrospective single-center Australian study, median patient survival was longer than in our study at 21 months,^18^ whereas in another, this value was similar to our findings, at a median survival of 14 months.^19^ Although the present study only included people above the age of 65 years, the age ranges in these other cohorts were similar.^18^^,^^19^ This study did not compare to the management of kidney failure with dialysis.

The finding of an association between living situation and survival is important as living situation is easily determined, the association is consistent with what clinicians might expect, and the association is underexplored in the literature—it has not been studied in other published cohorts.^9^, ^10^, ^11^, ^12^ Systematically documenting living status and considering it during discussions of prognosis and shared decision-making may help provide more forward-looking care. A living situation may itself be a reflection of the patients’ frailty, their functional status, or social support. Further studies to examine contributions to this predictor may be helpful.

In this study, there was no association between survival and comorbidities such as ischemic heart disease, angiotensin-converting enzyme/angiotensin receptor blocker use, or heart failure. Although another study identified no association with age or comorbidity burden,^18^ another found that heart failure was associated with worse survival,^19^ particularly among those aged >80 years.^18^^,^^19^

The finding of weakness and poor mobility as the predominant symptoms has been observed in another Australian study,^19^ whereas a different study found drowsiness and dyspnea to be the most common in a cohort with similar baseline characteristics to the people in our study.^20^ In our study, there was no major change in the median IPOS-Renal scores from baseline and follow-up, which could be due to a number of factors, including interventions by the KSC service, the death of people with the greatest symptom burden, or a small sample size skewing the results.

The median of 2 hospitalizations per patient observed in our study was similar to that observed in a large meta-analysis of people receiving conservative kidney care, which found 1-2 hospitalizations per person-year.^11^ Several studies have identified a significantly lower risk of hospitalization with conservative kidney care than with dialysis.^10^^,^^12^^,^^19^

The strengths of the study include a comprehensive assessment of a range of patient variables, which were well captured by the electronic medical record, and the assessment of patient-important outcomes. Our study was limited by its retrospective nature; a small sample size for certain results, such as among those with an eGFR of < 5 mL/min/1.73 m^2^ or those with symptom scores after 3 years; and the effects of analyzing a single center. Confounding may have occurred because of unmeasured variables such as smoking status or frailty and a lack of multivariable adjustment for measured and unmeasured variables.

In conclusion, the study provided valuable insights into outcomes for people with kidney failure managed without dialysis, including trends in life expectancy, the significance of the baseline living situation on prognosis, and the potential symptom burden to help guide clinicians and patients facing the uncertainty of this clinical scenario.
